# Editorial: Machine Learning Used in Biomedical Computing and Intelligence Healthcare, Volume II

**DOI:** 10.3389/fgene.2022.850667

**Published:** 2022-05-09

**Authors:** Honghao Gao, Ying Li, Zijian Zhang, Wenbing Zhao

**Affiliations:** ^1^ Shanghai University, Shanghai, China; ^2^ Zhejiang University, Hangzhou, China; ^3^ The University of Auckland, Auckland, Netherlands; ^4^ Cleveland State University, Cleveland, OH, United States

**Keywords:** machine learning, biomedical computing, intelligence computing, healthcare, data processing and analysis

Biomedical intelligence, especially precision medicine, is considered as one of the most promising directions in healthcare development. However, these technologies have also brought new challenges and issues. In 2021, this research topic was supported by Frontiers again and included three collaborating journals, namely, Frontiers in Genetics, Frontiers in Public Health, and Frontiers in Bioengineering and Biotechnology. Ten papers were accepted for publication from 18 open submissions. A summary of these accepted papers is outlined below.

In the paper entitled “Surveillance Strategy for Barcelona Clinic Liver *Cancer* B Hepatocellular Carcinoma Achieving Complete Response: An Individualized Risk-Based Machine Learning Study” by Qi-Feng Chen et al., the authors proposed a retrospective, real-world study on surveillance strategies for Barcelona Clinical Liver *Cancer* stage B hepatocellular carcinoma (BBHCC) patients with complete response (CR) after curative treatment to support clinical decision making. The data in this paper were collected from the *Cancer* Center of Sun Yat-sen University, part of which were used as training dataset and the rest as validation dataset. The random survival forest method was applied to calculate the disease progression hazard per month, and follow-up schedules were arranged to maximize the capability of progression detection at each visit. The primary endpoint of the study was the delayed-detection months for disease progression. The proposed new surveillance schedule may provide a new perspective concerning follow-up for BBHCC patients with CR.

In the paper entitled “Deep Learning Assisted Neonatal Cry Classification via Support Vector Machine Models” by Ashwini K et al., the authors presented a short-time Fourier transform (STFT) technique to transform the neonatal cry auditory signals into a spectrogram image. The deep convolutional neural network (DCNN) was used to extract features for neonatal cry classification with spectrogram images as input. Support vector machine (SVM) was used as the classifier based on the features automatically extracted. The study showed that the combination of using DCNN-based feature extraction and using the SVM classifier provided promising results. Specifically, the RBF kernel provided the highest classification accuracy of infant cry.

In the paper entitled “Identification of Key mRNAs as Prediction Models for Early Metastasis of Pancreatic *Cancer* Based on LASSO” by Ke Xue et al., the authors proposed a risk-scoring model to identify potentially robust predictors of metastasis through a minimal number of genes. Enrichment analysis of differential gene expression from multiple datasets was used to gain insight into the mechanism of pancreatic cancer metastasis. The study showed that six Epithelial-Mesenchymal Transition related genes can be used to reliably predict pancreatic cancer metastasis, assess clinical outcomes, and facilitate future personalized treatment for patients with ductal adenocarcinoma of the pancreas.

In the paper entitled “An Intelligent Control Model of Credit Line Computing in Intelligence Health-Care Systems” by Rong Jiang et al., the authors introduced a dynamic permission intelligent access control model that incorporated a credit line calculation to reduce the risk of patient privacy leakage when medical data are accessed. More specifically, the credit limit and credit interval according to the authorization rules were matched to control the access intelligently. The proposed method was validated using real patient data provided by a Grade-III Level-A hospital in Kunming, China.

In the paper entitled “Recurrence Risk of Liver *Cancer* Post-hepatectomy Using Machine Learning and Study of Correlation With Immune Infiltration” by Xiaowen Qian et al., the authors introduced an mRNA-based model to predict the risk of recurrence after hepatectomy for liver cancer and explore the relationship between immune infiltration and the risk of recurrence after hepatectomy. This paper investigated gene expression profiles of liver cancer patients, and selected 18 mRNAs as biomarkers for predicting the risk of recurrence of liver cancer using a machine learning method. The authors evaluated the immune infiltration of the samples and conducted a joint analysis of the recurrence risk of liver cancer. These findings are helpful for early detection, intervention, and the individualized treatment of patients with liver cancer after surgical resection. The scientific novelty in this paper is that it uses Machine Learning to forecast the recurrence risk of liver cancer post-hepatectomy.

In the paper entitled “Two-stage Deep Neural Network via Ensemble Learning for Melanoma Classification” by Jiaqi Ding et al., the authors presented an ensemble method that can integrate different types of classification networks for melanoma classification. U-net was used to segment the lesion area of images to generate a lesion mask, thus resizing images to focus on the lesion. In addition, a squeeze-excitation block was added to models to emphasize the informative features. Then, five classifiers were used to classify dermoscopy images. Finally, the proposed ensemble network was used to integrate the classification results from the five classifiers. The proposed classification framework was validated using the ISCI 2017 challenge dataset with good result. The scientific novelty in this paper is that it uses Deep Neural Network to make full use of the rich and deep feature information of images for melanoma classification.

In the paper entitled “Combining Polygenic Risk Score and Voice Features to Detect Major Depressive Disorders” by Yazheng Di et al., the authors proposed a biomarker of major depressive disorders (MDD) by combining the polygenic risk scores (PRSs) and voice features. Data in this study were collected from 3,580 women with recurrent MDD and 4,016 healthy people. PRS was constructed as a gene biomarker by p value-based clumping and thresholding. Voice features were extracted using the i-vector method. n the paper entitled “Contextualizing Genes by Using Text-Mined Co-Occurrence Features for *Cancer* Gene Panel Discovery” by Hui-O Chen et al., the authors introduced a pipeline that can contextualize genes by using text-mined co-occurrence features. Biomedical Natural Language Processing (BioNLP) techniques were used for literature mining in the cancer gene panel. The produced cancer gene panel was validated with the mutational landscape of different cancer types. The receiver operating characteristic (ROC) curve analysis confirmed that the neural net model has a better prediction performance. The key insight is that the use of text-mined co-occurrence features can contextualize each gene. This study examined several existing gene panels and demonstrated that part of the gene panel set can be used to predict the remaining genes for cancer discovery.

In the paper entitled “Classification of Diabetic Foot Ulcers Using Class Knowledge Banks” by Yi Xu et al., the authors presented a method that used class knowledge banks (CKBs) consisting of trainable units to effectively extract and represent class knowledge, and to better utilize the knowledge in the training data. Each unit in a CKB is used to compute similarity with a representation extracted from an input image. The averaged similarity between units in the CKB and the representation can be regarded as the logit of the considered input. In this way, the prediction depended not only on input images and trained parameters in networks, but also on the class knowledge extracted from the training data and stored in the CKBs. The experimental results showed that the proposed method effectively improved the performance of diabetic foot ulcers infection and ischemia classifications.

\In the paper entitled “Chronological Age Prediction: Developmental Evaluation of DNA Methylation-Based Machine Learning Models” by Haoliang Fan et al., the authors proposed a blood epigenetic clock in Southern Han Chinese (CHS) for chronological age prediction with machine learning algorithms. The correlation coefficient was analyzed for the experimental individuals to select five genes from a candidate set of nine age-associated DNA methylation (DNAm) biomarkers. The DNAm-based profiles of the CHS cohort were generated by the bisulfite targeted amplicon pyrosequencing (BTA-pseq) from 34 cytosine-phosphate-guanine sites of five selected genes. These four chronological age prediction models were evaluated using several machine learning algorithms, including stepwise regression, support vector regression, and random forest regression. The random forest regression appeared to achieve the best performence with a median absolute deviation of 1.15 years for the targeted cohort.

In conclusion, we would like to thank all the authors who submitted their original articles to our Research Topic. We highly appreciate the contributions of the reviewers for their suggestive comments. We would also like to acknowledge the guidance from the Editor-in-Chief and staff members of Frontiers.

